# Epidemiology of bovine tuberculosis in Tanzania: Systematic review and meta-analysis

**DOI:** 10.1371/journal.pntd.0014459

**Published:** 2026-07-06

**Authors:** Yohana Siyajali Anatory, Blandina T. Mmbaga, Beatus Lyimo, Joram Buza

**Affiliations:** 1 School of Life Sciences and Bioengineering, The Nelson Mandela African Institution of Science and Technology (NM-AIST), Arusha, Tanzania; 2 School of Agriculture, Mwalimu Nyerere University of Agriculture and Technology, Butiama, Tanzania; 3 Kilimanjaro Clinical Research Institute, Moshi, Kilimanjaro, Tanzania; 4 School of Medicine, KCMC University, Moshi, Kilimanjaro, Tanzania; Yale School of Medicine: Yale University School of Medicine, UNITED STATES OF AMERICA

## Abstract

**Introduction:**

Bovine tuberculosis (bTB) is an important animal disease in the world, with both economic and zoonotic implications. The disease is still endemic in developing world, such as Tanzania due to lack of rigorous bTB control measures. Despite its significant economic and public health importance in Tanzania, its epidemiological information is still inconsistent.

**Methods:**

To address this, we carried out a systematic review and meta-analysis of published articles on bTB epidemiology in Tanzania from 1993 to 2024. Eleven epidemiological themes were examined: prevalence, distribution, incidence, transmission, risk factors, reservoirs, diagnostics, outbreaks, surveillance, genetic diversity, and control measures. Data were synthesized using narrative analysis and random-effects meta-analysis where sufficient homogeneous data existed.

**Results:**

Thirty-eight studies met inclusion criteria, with studies addressing bTB prevalence exhibiting high heterogeneity (I² > 90%) and the pooled prevalence of 1.0% (95% CI: 0.01-0.02) in both livestock and wildlife. Mbeya and Iringa regions experienced the highest prevalence, whereas Dodoma and Lake Zone had the lowest prevalence. Large herd size, communal grazing, older cattle, and proximity to wildlife were consistent risk factors. African buffaloes were the most important wildlife reservoirs, sharing strains with cattle at the livestock-wildlife interface. The *Mycobacterium bovis* spoligotype SB0133 was the most common lineage. The tuberculin test and culture were the most frequently used diagnostic approaches. Outbreak, incidence and surveillance studies were scarce and fragmented.

**Conclusion:**

Although bTB is endemic in Tanzania, it has received little attention with geographical and thematic gaps still existing especially in incidence and surveillance. There is an urgent need for national strategy that integrates public health, livestock and wildlife systems. Also, molecular diagnostics, organized surveillance, outbreak investigations, and risk-based control measures should be among the priority areas.

## Author summary

Bovine tuberculosis (bTB) is an important disease that is known to be transmissible between animals and can be threat to people and their livelihoods especially in developing countries like Tanzania. While it is known to be present, the full picture of where it is, how it spreads, and its impact has been unclear. We conducted the first comprehensive review to bring together thirty-one years of research on this disease in Tanzania. We found that the overall infection rate in both livestock and wildlife is low (around 1%), but it hides significant hotspots, especially in regions where livestock and wildlife mix. Herd size, older animals, and grazing near wildlife interfaces are key risk factors. Our analysis revealed major gaps: there is almost no data on how often new infections occur (incidence) or on structured national surveillance programs. Also current control efforts are inadequate. Our work highlights the urgent need for a coordinated national strategy. Tanzania requires a unified plan that brings together veterinary, wildlife, and public health officials. Investing in better disease tracking and diagnostics will be crucial to protect animal health, safeguard communities, and support the livestock economy.

## Introduction

Bovine tuberculosis (bTB) is an animal disease caused chiefly by *Mycobacterium bovis*, characterized by tuberculous lesions in various organs particularly lungs. The disease affects a quite range of animals including livestock, wildlife as well as humans [1]. Bovine tuberculosis causes significant economic losses in livestock sector through reduced animal productivity, trade embargos and carcass/organ condemnations. International institutions such as WHO, WOAH, and FAO acknowledge bTB as an important zoonosis that requires a coordinated control strategy under a One-Health approach [2].

In sub-Sahara, bTB is still common with its prevalence estimated at 5.06% in cattle [3], underlining its significance in animal and public heath sectors in the region. However, its burden varies between countries, for example, the prevalence is estimated at 5.5% in Ethiopia, 2.94% in Nigeria and 6.8% in Zambia [4–6]. This variation in prevalence could be due to differences in implementation degree of control measures, production methods, geographic settings and diagnostic techniques.

Bovine tuberculosis in Tanzania seems to be endemic in regions which experience high livestock-wildlife contacts, in spite of the fact that its prevalence remains low in many parts of the country [7]. Based on researches done in Tanzania, the bTB burden is reported to range between 0.2% and 13.3% [8]. Animals in pastoral system appear to have high burden of the disease, probably due to sharing grazing lands and water sources, livestock movement, and proximity to wildlife areas [9]. Although the Tanzanian Animal Diseases Act classifies bTB as notifiable, its control and surveillance mechanisms are still not fully operational [10].

Like in other developing countries, bTB studies in Tanzania are dispersed and differ in approaches, geographic scope and diagnostic technique [11]. Because of this, it is challenging to fully understand the bTB epidemiology, this makes it difficult to develop data-based control, surveillance measures and policies as well as make well-informed decisions [8].

In light of the highlighted gaps in epidemiological data and its implications to livestock and public health sectors, a thorough synthesis of current epidemiological evidence of bTB in Tanzania is of paramount importance. The purpose of this review is to compile data on the epidemiology of bTB in Tanzania using eleven sub-themes: prevalence, distribution, incidence, transmission, risk factors, reservoirs, diagnostics, genetic diversity, outbreaks, surveillance, and control measures. This will guide Tanzanian policymakers, researchers, and other key stakeholders in prioritizing data-driven research, control and surveillance strategies.

## Methods

### Literature search strategy

Search strategies were established based on keywords related to bTB epidemiology in Tanzania. The formulated search query encompassed three core concepts which are; the disease, the host and the geographic location. Some of the keywords searched include “bovine tuberculosis”, cattle, and Tanzania. The search was done on the following databases: PubMed, Scopus, AJOL, Google Scholar and Embase. We manually skimmed the reference lists of the eligible articles to identify any potentially relevant studies. Relevant titles identified from reference lists we subjected to the same screening process as those derived from databases. Lastly, a bibliography of the included articles was shared with the systematic review team.

The search query was synthesized based on previous reviews on bovine tuberculosis epidemiology, which helped in identifying relevant databases and search terms. The specific search query for each database was synthesized and finalized by the project team with the help of a Life Sciences Librarian. The PubMed search query was synthesized by the review team and cross-checked by another librarian. After ensuring that the finalized PubMed search strategy retrieved high proportion of relevant studies was tailored and adapted to the other four databases. A search strategy of each database is included in S1 Table.

### Inclusion and exclusion criteria

We included original research articles that (1) reported on single or more bTB epidemiological elements namely; prevalence, incidence, surveillance, distribution, risk factors, transmission, reservoirs, control measures, diagnostics, outbreaks and genetic diversity (2) were carried out entirely or in part in Tanzania from 1993 to 2024(3) were cross-sectional, case control, or cohort (including longitudinal) studies that reported data on bTB epidemiology at a district, regional, zonal or national level (4) were reported in the English language and done in either livestock, wildlife animals or both. Exclusion criteria included (1) any review and articles whose full text were unavailable; (2) those focusing on clinical trials and those done before the year 1993 and after 2024.

### Data extraction/collection

#### Data management.

A Microsoft Excel spreadsheet was used to manage all the retrieved studies and eliminate duplicates.

#### Selection process.

Studies were screened by their titles and abstracts, followed by the retrieval and screening of full-text articles based on the aforementioned inclusion and exclusion criteria. Two independent reviewers screened the articles and in case of any disagreements, discussion was used to settle these disagreements between reviewers and in some few instances a third reviewer was involved as tie-breaker. Reasons for any excluded article were documented. A flow chart of the PRISMA 2020 flow diagram for new systematic reviews was used to trace the overall screening process.

#### Data collection process.

This procedure involved two reviewers to extract data from the included studies. One reviewer extracted data from all selected articles, while the other reviewer cross-checked all articles for accuracy and independently extracted data from 30% of the articles. Discussion was used to settle disagreements between reviewers and in some cases a third reviewer was involved.

#### Data items.

Data relating to a) prevalence estimates b) incidence c) bTB surveillance d) distribution e) transmission f) *M. bovis* genetic diversity g) risk factors h) reservoirs i) control measures j) outbreaks were collected. Also, data on k) method of bTB diagnosis e) type and species (if available) of animal involved; l) region/zone of Tanzania where the study was conducted; and m) sample size used in the study were recorded

### Statistical analysis

Risk of bias assessment of individual studies was done by assessing the quality of the studies to be included in the meta-analysis, so that to assess the strength of the proof on measure effect of interest. This process was done during the data extraction process, where two independent reviewers were involved in this process. Any disagreements were resolved via discussion between reviewers or by involving a third reviewer. The *Risk of Bias Tool* for assessment of the risk of bias for population-based studies [12] was used in appraising possible risk of bias.

Each of the included studies was assigned to an epidemiological parameter(s) it addressed. For epidemiological parameters with sufficient homogeneous quantitative data, in this case prevalence, genetic diversity and risk factors; a meta-analysis was done to estimate pooled effect (s) by a random-effects model. However, the remaining epidemiological parameter(s) with sparse, heterogeneous, or insufficient quantitative data, a structured narrative synthesis was conducted.

Meta package of R software (V 4.4.0) was employed for meta-analysis, the statistical significance of the epidemiological data was determined at 95% CI. Forest plots were generated displaying bTB epidemiological parameter corresponding with 95% CIs for each study. The overall random-effects of pooled effect size (for each epidemiological parameter with sufficient homogeneous quantitative data) with their CIs are reported in this paper. Since the true effect was likely to differ between studies [13], the random-effects model by DerSimonian and Laird method [14] was employed for variance estimation.

To avoid skewed results by grouping very different studies, the selected prevalence studies were further sub-grouped based on host species (livestock vs. wildlife), and geographic regions (administrative zones or regions with sufficient data). For easier interpretation, risk factors were analyzed based on three groups: environmental, animal-level and herd-level risk factors. Where multiple risk factors were extracted from the same study, these were treated as separate data points for meta-analysis. Raw reported effect sizes of bTB epidemiological parameter served as basis for computing the pooled effect size of that parameter.

Where possible, the pooled effect size of each parameter and the 95% Cis of studies with similar characteristics were calculated based on geographical regions and/or animal classification (livestock or wildlife). Where possible, parameter effect size was broken down by the method of bTB diagnosis, and non-overlapping confidence intervals were considered an evidence of differences that are statistically significant.

Heterogeneity tests were carried out to determine the degree of variation among the selected research. Since the review pooled highly heterogeneous studies, random effects models were chosen a priori to address the anticipated heterogeneity. To test the null hypothesis which assumed all studies had the same effect size [13], the Q-statistic was used. Furthermore, to determine the proportion of observed variance that reflected genuine effect sizes rather than sampling error, the Higgins (I²) statistic was utilized, which was presented as a percentage [15]. The I² statistic indicated what proportion of variance remained after removing sampling error. Higgins and colleagues [15] proposed preliminary criteria for I², with values below 25% deemed low, 50–75% as moderate, and above 75% as high. Furthermore, because I² did not offer absolute variance dispersion, T² was estimated based on the observed effects to determine the real variance dispersion.

### Publication bias

Visual examination of funnel plots was employed to evaluate evidence of publication bias for epidemiological parameter with sufficient quantitative data. The Egger regression asymmetry test evaluated the extent of bias and its effect on the results. The funnel plot was plotted with effect size on the X-axis and sample size on the Y-axis. Any asymmetric distribution of studies around the mean effect size provided the first indication of the presence of publication bias [13].

The trim-and-fill technique was used to remove extreme outliers (small studies) if publication bias was found. The missing studies were then added (“filled”) by duplicating the removed studies on the other side. These “filled” studies represented hypothetical missing studies that could have contributed to the observed asymmetry. This was followed by a re-calculation of the effect size until the funnel plot became symmetrical, thereby correcting the observed publication bias [16,17].

## Results

### Study selection and characteristics

**Fig 1:**
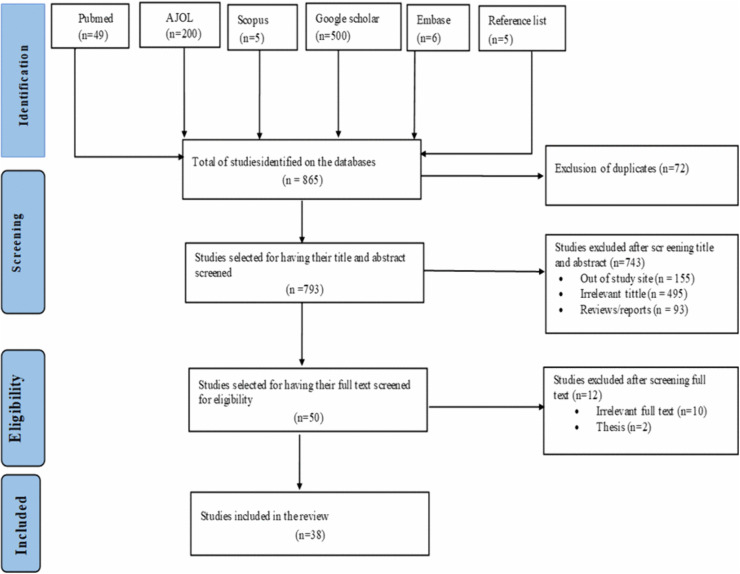
The PRISMA flow chart of studies. **Note:** The figure shows the results of the search and selection process, 865of records were identified in the search of five databases and reference list, 72 articles were duplicates, 743 were eliminated during title and abstract screening and 6 more articles were removed during full text screening. Only 38 articles met the inclusion criteria to be included in the review.

A total of 865 articles were retrieved, of those 72 were duplicates. The remaining 793 titles and abstracts were screened, fifty articles remained for their full-texts assessment. Thirty-eight articles met the inclusion criteria and were included in this review (Fig 1).

The majority of articles employed cross-sectional design and only a small number of them involved more countries than Tanzania. A total of thirteen regions were represented by reviewed articles, led by Morogoro (n = 12 studies), Arusha (n = 9), and Iringa (n = 6). Other regions such as Dar es Salaam, Coast, Dodoma, Manyara, Mbeya, and Tanga were represented by two studies each. One study was conducted in multiple Lake Zone regions (Shinyanga, Mwanza, Kagera, Mara), whereas two studies only specified ecological zones rather than regions. Thirty-one studies were done in livestock, with cattle leading as the primary target. Six studies focused exclusively on wildlife, three were done in both cattle and wildlife, and two apart from animals included environmental samples (soil and dust) (S2 Table).

### Prevalence

**Fig 2:**
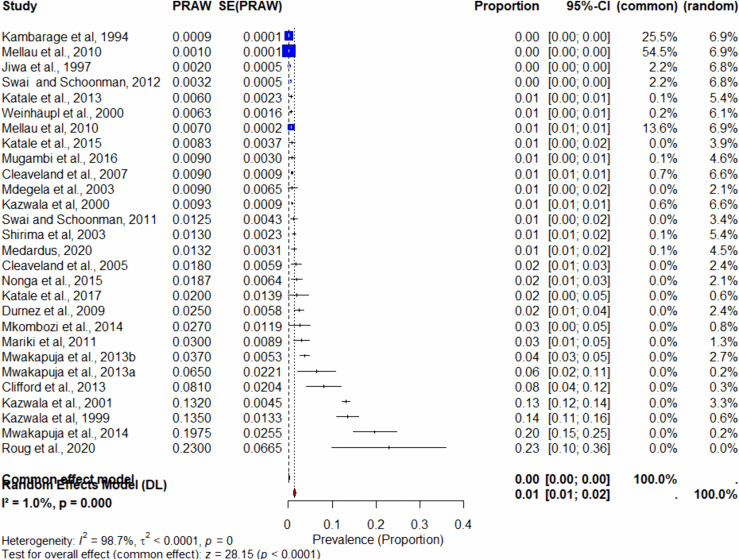
Forest plot of prevalence studies in both livestock and wildlife. **Note:** the figure shows thirty-three prevalence studies in animals (livestock and wildlife). The forest plot displayed the overall pooled prevalence of 0.01 (95% CI: 0.01-0.02), with a highly significant test for overall effect (z = 28.15, p < 0.0001). Heterogeneity assessment by random effects model showed significant variability between studies (*I*² = 98.7%, *τ*² < 0.0001, p < 0.001).

Thirty-three studies included prevalence data, the reported prevalence ranged from 0% to 19.75%, influenced by region, production system, and diagnostic method. The forest plot displayed the overall pooled prevalence of 0.01 (95% CI: 0.01-0.02), with a highly significant test for overall effect (z = 28.15, p < 0.0001). Heterogeneity assessment by random effects model showed significant variability between studies (*I*² = 98.7%, *τ*² < 0.0001, p < 0.001) (Fig 2), suggesting this overall estimates should be interpreted with caution due substantial heterogeneity. With exception of a few, most of the prevalence studies observed low prevalence rates (<5%).

**Fig 3:**
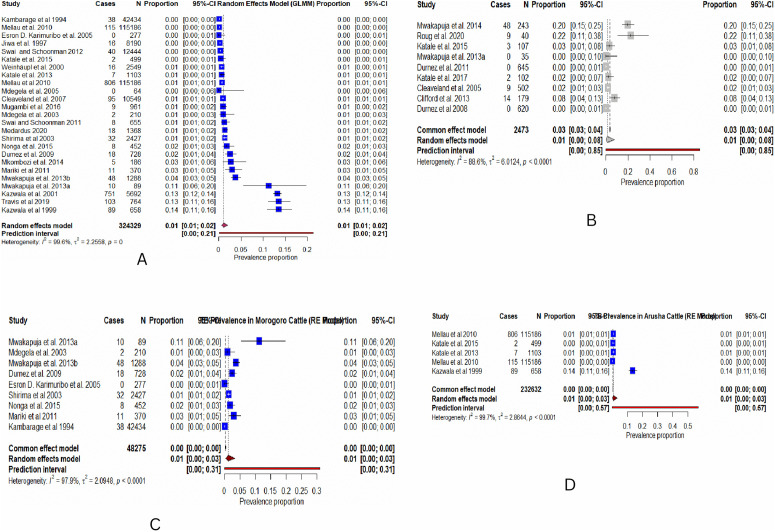
Subgroup forest plot of prevalence studies in (A) cattle (B) wildlife (C) Morogoro (D) Arusha. **Note**: (A) shows a forest plot of twenty-five prevalence studies in cattle, the studies show high heterogeneity (*I²* = 99.6%, τ² = 2.26) with the random effects model pooled prevalence of 1.13% (95% CI: 0.01-0.02). (B) shows a forest plot of nine prevalence studies in wild animals which showed high heterogeneity in bTB prevalence across wildlife populations (*I²* = 88.6%, τ² = 6.01) with the random effects model pooled prevalence of 1.35% (95% CI: 0.00-0.07). (C) is the forest plot of nine prevalence studies done in Morogoro, the studies showed high heterogeneity (*I²* = 97.9%, τ² = 2.095, p < 0.0001) in bTB prevalence across Morogoro cattle populations with the random-effects model pooled prevalence of 1% (95% CI: 0.00-0.03). (D) shows the forest plot of five prevalence studies in cattle done in Arusha, the studies are extremely heterogeneous (*I²* = 99.7%, p < 0.0001) with a pooled prevalence estimate of 1% (95% CI: 0.00–0.03).

Subgroup analysis of prevalence studies was done based on host species (cattle and wildlife) and geographical region. Twenty-six prevalence studies addressed prevalence in cattle, but only twenty-five studies were included in the heterogeneity assessment. These studies in cattle high heterogeneity (*I²* = 99.6%, τ² = 2.26) with the random effects model pooled prevalence of 1.13% (95% CI: 0.01-0.02) (Fig 3A). On the other hand, nine studies reported bTB prevalence in wild animals and showed high heterogeneity as well (*I²* = 88.6%, τ² = 6.01). The random effects model of studies in wildlife estimated a pooled prevalence of 1.35% (95% CI: 0.22%-7.83%) (Fig 3B).

Only Morogoro (nine studies) and Arusha (five studies) were included in subgroup analysis by regions, because other regions did not have enough prevalence studies for meta-analysis to be conducted. Analysis of the nine studies done in Morogoro showed high heterogeneity (*I²* = 97.9%, τ² = 2.095, p < 0.0001) with an estimated pooled prevalence of 1% (95% CI: 0.00-0.03) (Fig 3C) across Morogoro cattle populations. Whereas, the five studies conducted in Arusha showed extremely high heterogeneity (*I²* = 99.7%, p < 0.0001) with a pooled prevalence estimate of 1% (95% CI: 0.00–0.03) (Fig 3D) across Arusha cattle populations.

### Distribution

**Fig 4:**
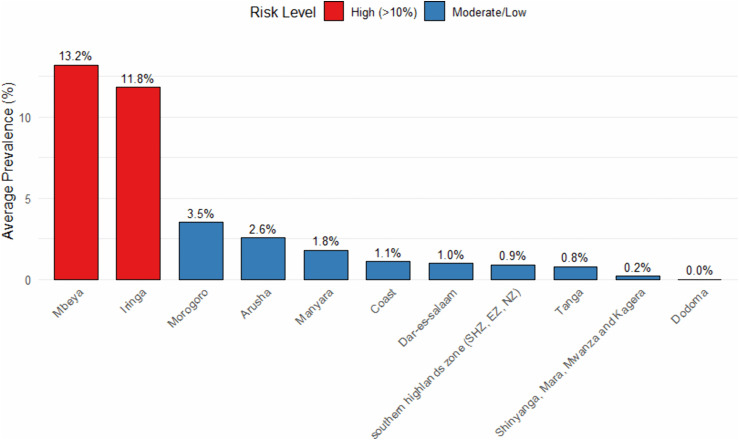
Bar chart of average bTB prevalence across regions in Tanzania. **Note**: The figure shows a bar chart of average bTB prevalence in eleven Tanzanian regions, the red colour represents high risk and blue colour represents moderate/low risk. Mbeya and Iringa showed high bTB risk with average prevalence of 13.2% and 11.8% respectively. The remainders showed either moderate or low bTB risk.

A total of thirty-three studies addressed bTB distribution across Tanzania, with Iringa (11.8%) and Mbeya (13.2%) recording high average prevalence compared to other regions. Moderate prevalence estimates were observed in Morogoro (3.5%) and Arusha (2.6%), suggesting localized transmission risks. Central Zone (Dodoma) and Lake Zone (Shinyanga, Mara, Mwanza and Kagera) had minimal cases of 0.0% and 0.2% respectively. Markedly, high prevalence in Mbeya stems from a single 2001 study (13.2%), whereas high average prevalence in in Iringa is influenced by extreme values from 2020 (23%) and 2019 (13.5%) (Fig 4). Regions near national parks (Serengeti, Ruaha and Mikumi) and game reserves recorded elevated bTB prevalence. As also noted, Lake and Western Zone regions were heavily underrepresented in retrieved articles. These estimates should be interpreted with caution, because the small number of studies and variable sample sizes tend to limit precision. The apparent absence of bTB in Dodoma for example, maybe a reflection of limited sampling rather than true absence. Also, the observed high average prevalence in Mbeya (13.2%) comes from a single 2001 study (n = 5692 cattle; 95% CI: 12.32-14.08%), and the estimate in Iringa (11.8%) is influenced by two studies reporting 23% (n = 40; 95% CI: 9.96-36.04%) and 13.5% (n = 764; 95% CI: 11.1-15.9%).

### Incidence

No study among the included articles addressed incidence of bovine tuberculosis in Tanzania.

### Risk factors

**Fig 5:**
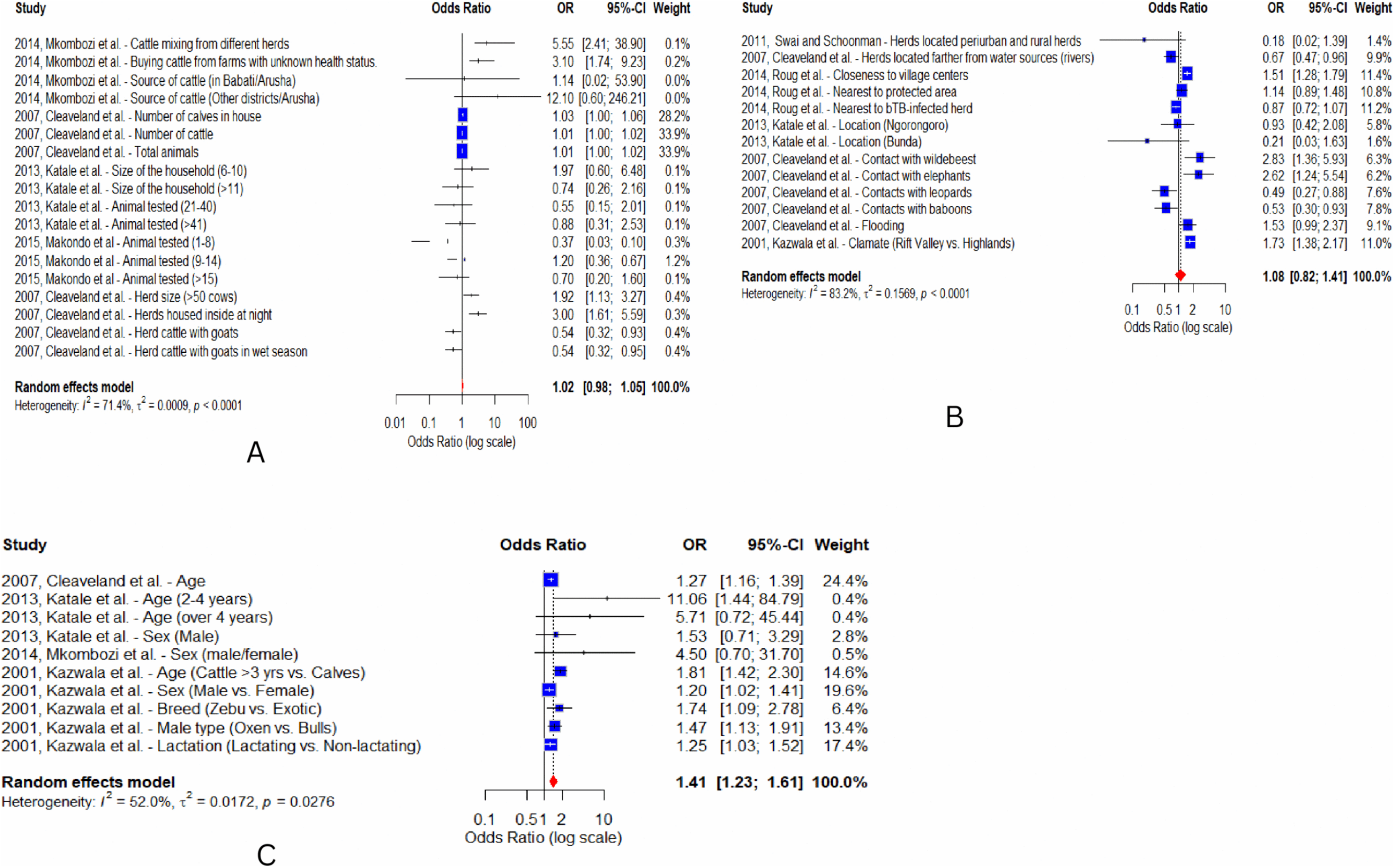
Forest plot of (A) herd-level factors (B) environmental factors (C) animal-level factors. **Note**: (A) shows forest plot of studies on herd-level risk factors, the studies showed moderate heterogeneity (*I²* = 71.4%, τ² = 0.0009) with overall pooled OR of 1.02 (95% CI: 0.98-1.05). (B) shows a forest plot of studies on environmental factors, the studies showed high heterogeneity (*I²* = 83.2%, τ² = 0.1569) with an overall OR of 1.08 (95% CI: 0.82-1.41). (C) shows a forest plot of studies on animal-level risk factors, the studies showed moderate variability in effect sizes (*I²* = 52.0%, τ² = 0.0172) with an overall OR of 1.41 (95% CI: 1.23-1.61).

Only five studies reported bTB risk factors in animals. However, each risk factor was treated as a study during analysis, bringing the total number of studies to thirty-eight. This approach may have violated the assumption of independence, and the results should therefore be interpreted as exploratory rather than confirmatory. To make meta-analysis easy, risk factors were grouped into; environmental, animal-level and herd-level risk factors. Herd-level risk factors (*I²* = 71.4%, τ² = 0.0009) (Fig 5A) and environmental factors (*I²* = 83.2%, τ² = 0.1569) (Fig 5B) showed substantial heterogeneity, suggesting significant variability in effect sizes between studies. In contrast, animal-level risk factors demonstrated moderate heterogeneity (*I²* = 52.0%, τ² = 0.0172) (Fig 5C). Overall pooled odd ratios were OR=1.08 (95% CI: 0.82-1.41), OR=1.41 (95% CI: 1.23-1.61) and OR=1.02 (95% CI: 0.98-1.05) for environmental, animal-level and herd-level risk factors respectively. Older cattle (>3 years) were more likely to test positive. Herds under communal grazing, had significantly higher infection rates compared to herds under zero-grazing system. Herd size and interaction with wildlife were identified to be associated with bTB transmission. Also, seasonality was identified to have influence on bTB exposure, as shown by a study in Ruaha where bTB prevalence was high (17.8%) in the dry season compared to (8.9%) in wet season (p = 0.0004).

### Transmission

Fourteen studies reported on bTB transmission dynamics of bTB between livestock to livestock, livestock to wildlife and vice versa with inhalation of aerosols from infected animals as the primary pathway. Studies confirmed presence of transmission between livestock and wild animals by isolation of related *M. bovis* strains from cattle and buffalo such as SB0133. Transmission was also associated with sharing grazing and watering points, as well as movement of untested animals.

### Reservoirs

Nine studies reported on bTB reservoirs, where African buffaloes (Syncerus caffer) were most reservoirs of the disease particularly in Ruaha, Mikumi and Serengeti ecosystems. This was supported by identical *M. bovis* strains (spoigotypes) isolated from both buffalo and cattle at livestock-wildlife interfaces. Results from other wildlife animals such as warthogs, antelopes, and giraffes were inconclusive. Also, a study in small wild mammals reported no *M. bovis* but only non-tuberculous mycobacteria.

### Genetic diversity

**Fig 6:**
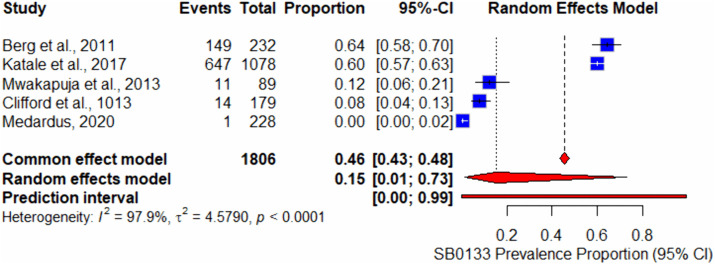
Forest plot of SB0133 prevalence studies. **Note:** The figure shows four studies on prevalence of SB0133 spoligotype in both livestock and wildlife. The studies showed high heterogeneity (*I²* = 97.9%, p < 0.0001) with a pooled prevalence of 0.15 (95% CI: 0.01-0.73).

Six studies reported on genetic diversity of *M. bovis* in Tanzania, SB0133 led as the prevalent spoligotype with an estimated pooled prevalence of 0.15 (95% CI: 0.01-0.73) in both livestock and wildlife. The forest plot of SB0133 prevalence studies showed significant heterogeneity (*I²* = 97.9%, p < 0.0001), with point prevalence ranging from 0.00 to 0.64 (Fig 6). The prevalence of other important spoligotypes namely; SB0425, SB1446, and SB1467 varied between regions. For instance, SB1467 was recorded in 62.5% of cattle isolates in Mikumi-Selous, whereas SB1446 was recorded in 7.1%. Detection of SB2190 and SB2290 stresses regional genetic variability of *M. bovis*, with some of its strains developing into clonal complexes such as Af2 in East Africa. One study reported in non-standard spoligotype names found 13 distinct spoligotypes (spA-spM) with 79% genetic relatedness.

### Diagnostics

Thirty-seven studies described diagnostic techniques (Table 1). The tuberculin test was the most widely used diagnostic technique in twenty-one studies, among these eighteen studies applied the Single Intradermal Comparative Tuberculin Test (SCITT) and three studies used the Single Intradermal tuberculin test (SITT). SCITT was a sole test in 11 studies, but combined with other tests in seven studies. Only one study used SITT as a sole test. Culture (11 studies) and PCR (7 studies) served as key confirmatory methods. Spoligotyping, post-mortem inspection, and gamma interferon assays were averagely used each in 5 studies. Less used methods included ELISA/EIA, RFLP, Deletion typing, Sequencing, BovidTB Stat-Pak and MIRU-VNTR.

**Table 1:** Frequency of diagnostic methods across included studies.

Study	Diagnostic method														
	*SITT*	*SCITT*	*Culture*	*PCR*	*Spoligotyping*	*PM inspection*	*γIFN*	*ELISA/EIA*	*RFLP*	*Deletion typing*	*Microscopy*	*Phenotypic techniques*	*Sequencing*	*BovidTB Stat-Pak*	*MIRU-VNTR*
*1. Cleaveland et al., 2017*		*	*												
*2. Mwakapuja et al., 2013a*			*		*					*					
*3. Kazwala et al., 2001*		*													
*4. Mwakapuja et al., 2013b*		*													
*5. Shirima et al.2003*		*													
*6. Kazwala et al., 2006*					*				*						
*7. Mugambi et al., 2016*		*													
*8. Cleaveland et al., 2005*			*					*							
*9. Travis et al., 2019*				*											
*10. Weinhäupl et al., 2000*	*			*											
*11. Roug et al., 2014*		*													
*12. Clifford et al., 2013*			*	*	*		*								
*13. Swai & Schoonman, 2012*	*	*													
*14. Durnez et al.,b2011*			*	*											
*15. Berg et al., 2011*					*					*					
*16. Kazwala et al., 1999*		*													
*17. Komba et al., 2012*						*									
*18. Mwakapuja et al., 2014*		*					*							*	
*19. Katale et al., 2017a*					*										*
*20. Katale et al., 2017b*							*								
*21. Jiwa et al., 1997*		*													
*22. Roug et al., 2020*							*								
*23. Karimuribo et al., 2005*		*	*												
*24. Mdegela et al., 2004*		*	*												
*25. Swai & Schoonman, 2012b*	*														
*26. Mdegela et al., 2005*		*	*					*							
*27. Katale et al., 2013*		*													
*28. Durnez et al., 2009*		*	*	*											
*29. Kazwala et al., 1998*			*	*											
*30. Mariki et al., 2013*						*	*								
*31. Mkombozi et al., 2014*		*													
*32. Medardus et al., 2020*		*													
*33. Kazwala et al., 2000*		*													
*34. Durnez et al., 2008*			*	*							*	*	*		
*35. Kambarage et al., 1995*						*									
*36. Mellau et al., 2010*						*									
*37. Mellau et al., 2011*						*									
**38. TOTAL**	**3**	**18**	**11**	**7**	**5**	**5**	**5**	**2**	**1**	**2**	**1**	**1**	**1**	**1**	**1**

### Outbreaks

Only two studies documented confirmed bTB outbreaks. First study reported an outbreak in Kagera region of 2.12% linked to a cattle herd imported from Dar-es-Salaam. Whereas, the second study noted outbreak-resembling clusters in Ruaha, Iringa, during drought seasons because of increased mixing of herds at water sources.

### Surveillance

No study was explicitly done as part of structured surveillance. Most data came from cross-sectional research, not government monitoring. On the other hand, one study noted the lack of “One Health” surveillance approach, where animal TB programs are not in sync with human TB programs. Furthermore, no studies reported any use of national electronic databases or reporting tools in the country.

### Control measures

The reviewed studies identified meat inspection, movement control, and selective testing as commonly used control measures. No article reported presence of national bTB control strategy in Tanzania. Limitations towards effective bTB control include lack of compensation for culled animals, low farmer awareness, and inadequate veterinary staffing. Some other recommended control strategies were pilot testing of vaccines, improved stakeholder engagement and integrating bTB control into zoonotic disease programs for cost-effectiveness. Also, one study designed a “One Health” approach to comprehensively implement multidisciplinary actions against bovine and zoonotic TB.

### Publication bias

#### Prevalence.

**Fig 7:**
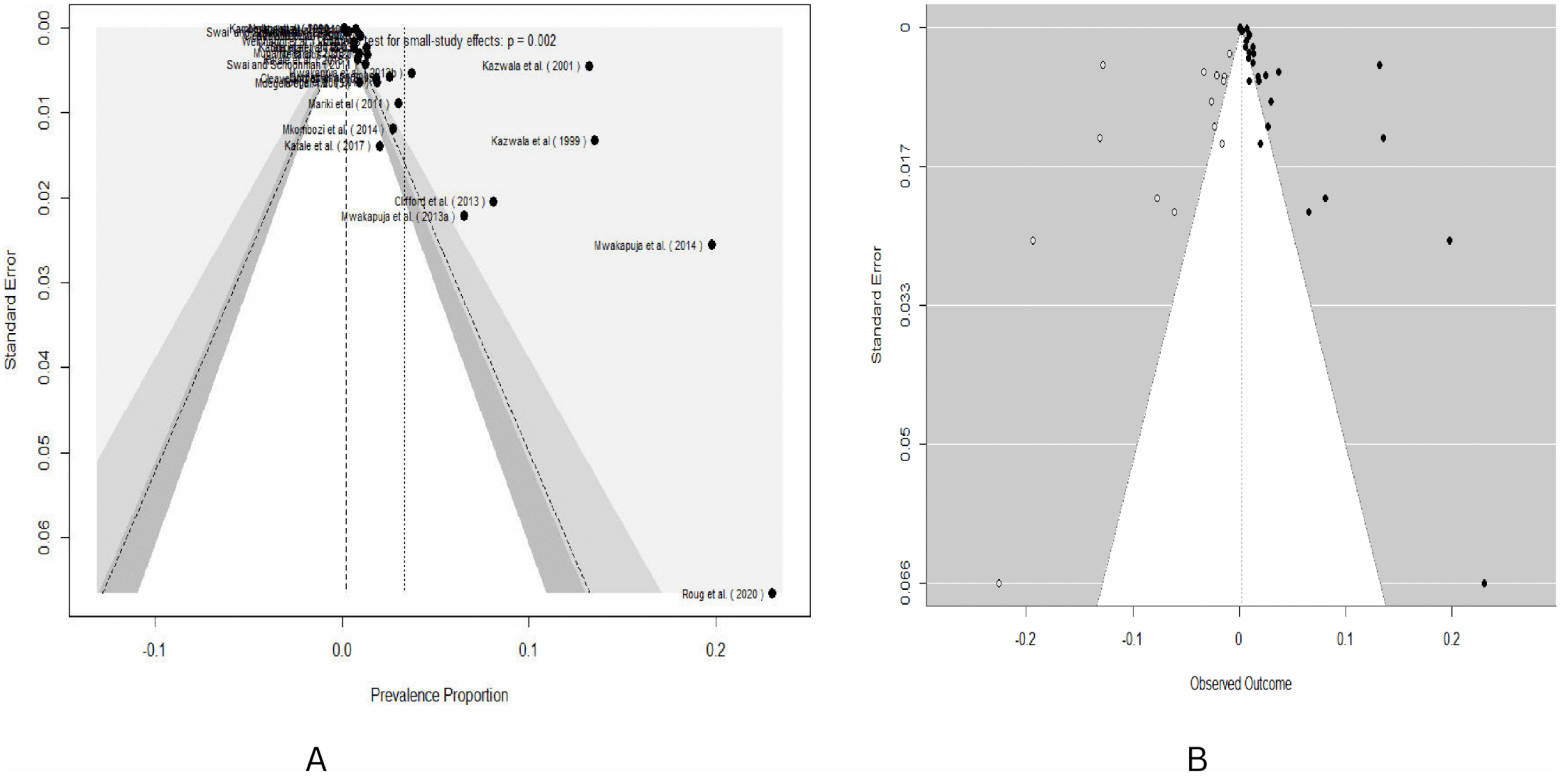
Funnel plot of (A) bTB prevalence studies (B) prevalence studies after trim-and-fill. Note: (A) shows funnel plot of prevalence studies in animas, the plot depicts noticeable asymmetry, with more studies concentrated on right side of the mean effect size. (B) shows the trim-and-fill adjusted funnel plot correcting publication bias, grey dots represent 14 imputed missing studies.

Publication bias assessment through funnel plot showed noticeable asymmetry, with more studies concentrated on the right side of the mean effect size, and Egger’s test confirmed presence of publication bias (p = 0.002) (Fig 7A). The trim-and-fill adjusted funnel plot corrected this asymmetry by imputing 14 potentially missing studies (Fig 7B).

**Fig 8:**
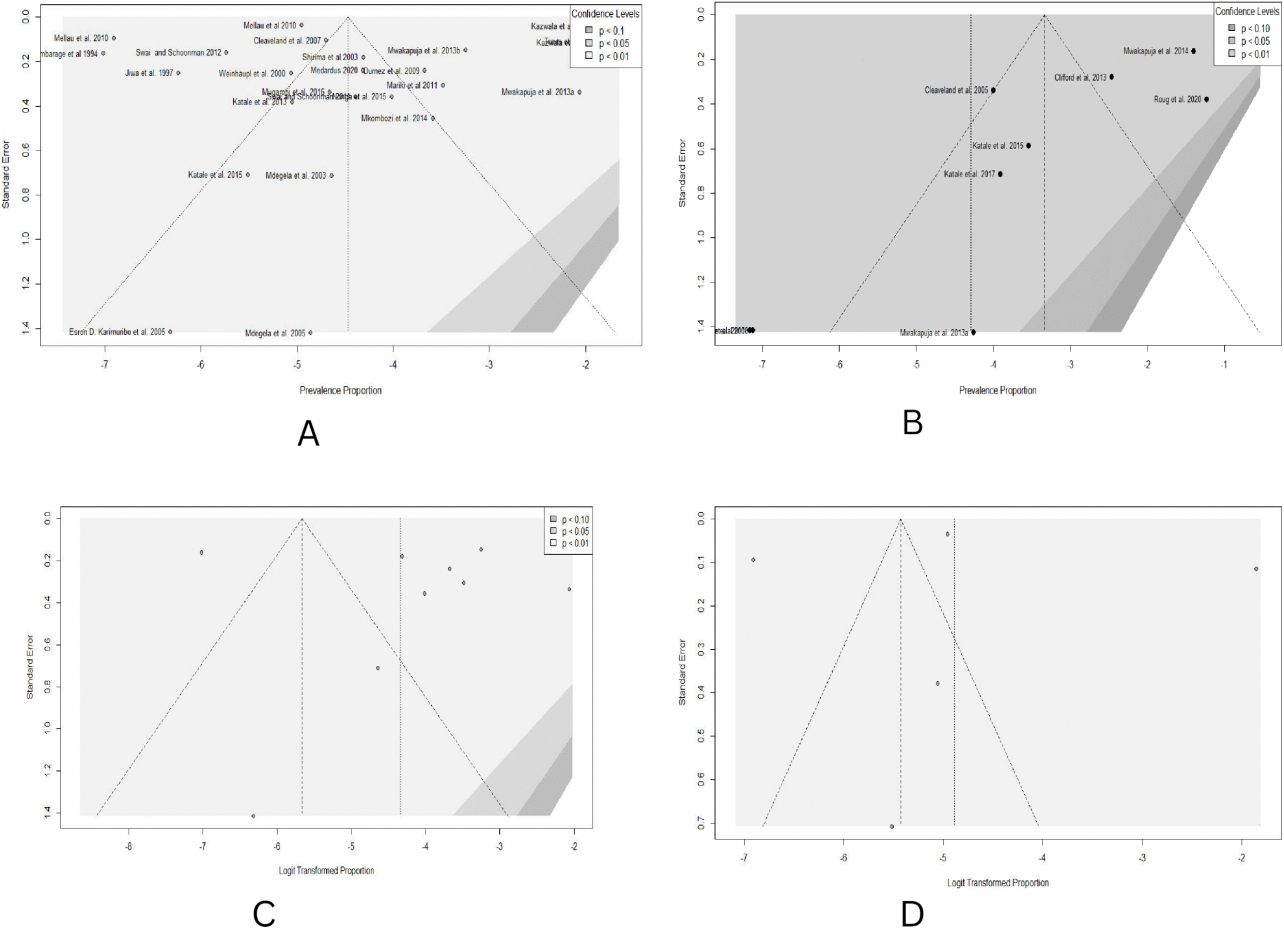
Funnel plot of prevalence studies in (A) cattle (B) wildlife (C) Morogoro (D) Arusha. **Note**: (A) shows funnel plot of prevalence studies in cattle, the studies are symmetrically distributed as evidence of absence of publication bias (t = -0.71, p = 0.487). (B) shows funnel plot of prevalence studies in wildlife, the studies showed asymmetrical distribution as evidence of presence of publication bias (t = -3.00, p = 0.02). (C) represents funnel plot of prevalence studies across Morogoro indicating no statistically significant evidence of publication bias and Egger’s test (t = 0.41, p = 0.691). (D) represents funnel plot of prevalence studies across Arusha demonstrating no significant bias publication bias (t = 0.15, p = 0.889).

The funnel plot and Egger’s test of prevalence articles in cattle revealed no significant bias in publication (t = -0.71, p = 0.487) (Fig 8A). However, both funnel plot and Egger’s test of prevalence studies in wildlife showed a significant publication bias (t = -3.00, p = 0.02) (Fig 8B). The trim-and-fill analysis imputed 5 studies raising the adjusted pooled estimate to 13.6% (95% CI: 3.0%-44.3%) due to extreme heterogeneity (*I²* = 93.5%, τ² = 8.61).

Funnel plot and Egger’s test showed no statistical significance of publication bias among prevalence studies from Morogoro (t = 0.41, p = 0.691, Fig 8C) and Arusha (t = 0.15, p = 0.889, Fig 8D).

#### Risk factors.

**Fig 9:**
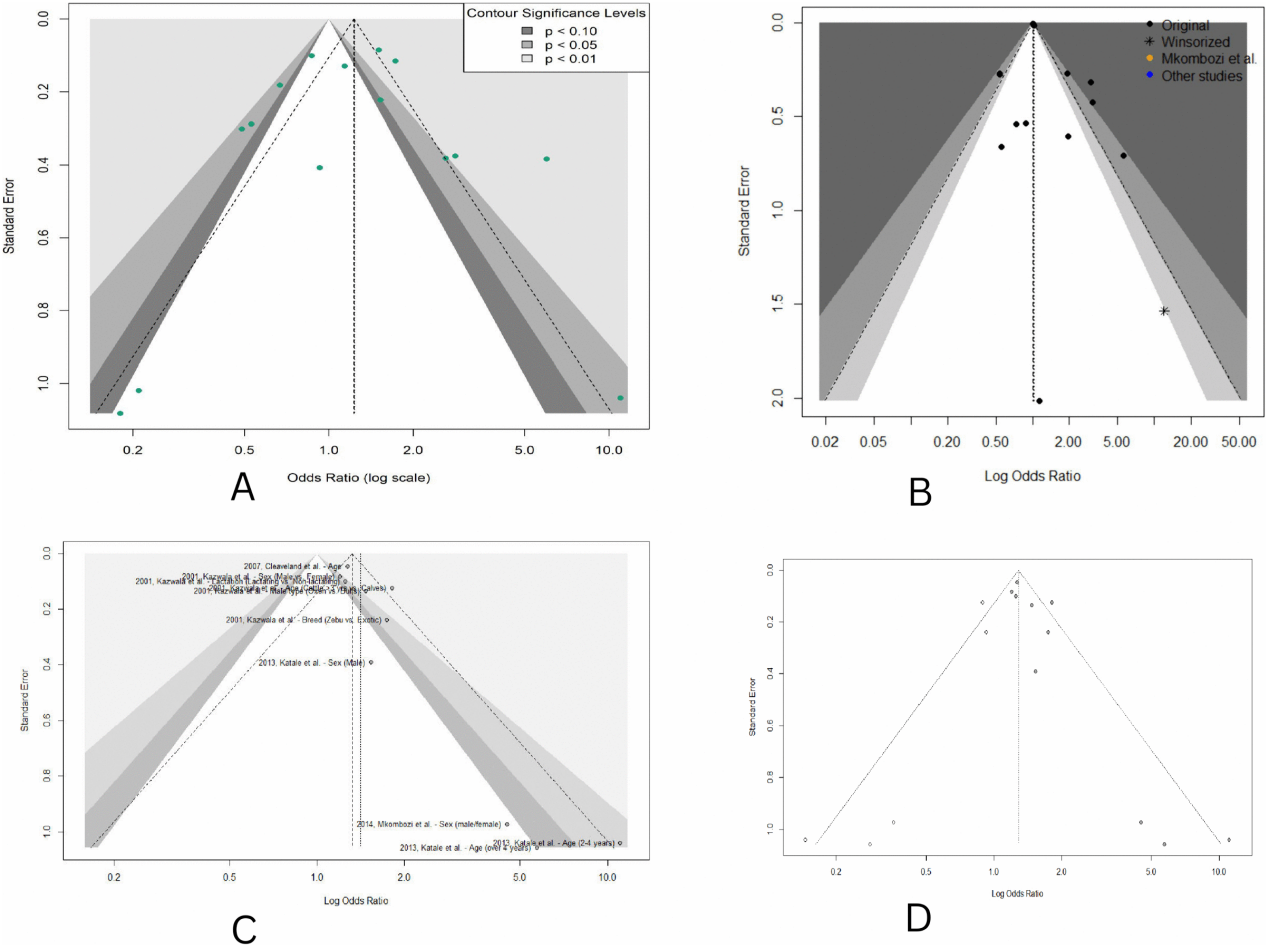
Funnel plot of studies on (A) environmental risk factors (B) herd-level risk factors (C) animal-level risk factors (D) animal-level risk factors after trim-and-fill. **Note:** (A) depicts funnel plot of studies on environmental-level risk factors revealing symmetrical distribution of studies hence absence of publication bias (p = 0.312). (B) shows funnel plot of studies on herd-level variables, the studies have symmetrical distribution as evidence of no significant publication bias (p = 0.2210). (C) shows funnel plot of studies on animal-level risk factors with asymmetrical distribution as evidence of presence publication bias (p = 0.0087). (D) shows the trim-and-fill adjusted funnel plot of studies on animal-level risk factors correcting asymmetry by imputing 5 potentially missing studies (represented by grey hollow dots).

Funnel plot examination and Egger’s test revealed no publication bias among studies on environmental risk factors (p = 0.312, Fig 9A) and herd-level risk factors (p = 0.2210, Fig 9B). On contrary, studies on animal-level risk factors showed publication bias (p = 0.0087, Fig 9C) and the trim-and-fill adjusted funnel plot corrected this asymmetry by imputing 5 potentially missing studies (Fig 9D).

#### Genetic diversity.

Results of publication bias on genetic diversity of *M. bovis* were unreliable because of small number of studies.

## Discussion

By drawing evidence from thirty-eight studies conducted between 1993 and 2024, this systematic review gives a comprehensive insight into epidemiology of bTB in Tanzania to date. The results show widespread detection of bTB across multiple geographic regions and host species. Its epidemiology is characterized by low overall prevalence, livestock-wildlife transmission, high regional variability and limited outbreak investigations. Additionally, diagnostic and surveillance systems continue to be fragmented, with ripple effects for both animal and human health. This discussion interprets the results within the context of existing literature, knowledge gaps, policy and One-Health implications for Tanzania and beyond.

The prevalence of bTB in Tanzania is extremely variable, with the range between 0% and 19.75%, but the overall prevalence estimate remains as low as 1%. These findings mirror previous reviews that reported ranges of 0.1–13.2% and 0.2–13.3% [8,18]. Nevertheless, the observed pooled prevalence seems to be markedly lower than that in Ethiopia (5.5%) [4] and sub-Saharan Africa (5.06%) [3]. The over reliance on the tuberculin skin test, which has moderate sensitivity [19], could be the reason for underestimation of true bTB prevalence in Tanzania. However, the 1% pooled estimate of bTB prevalence need to be interpreted with caution as it represents prevalence calculated from studies with high heterogeneity caused by diverse settings and characteristics. For this reason, subgroup analyses should be prioritized for inference instead.

In term of spatial distribution, bTB burden is concetrated in regions with livestock-wildlife interfaces such as Ruaha, Serengeti, and Mikumi, this underscores these interfaces as hotspots for bTB transmission [20]. The observed high prevalence in Mbeya (13.2%) and Iringa (11.8%) should be interpreted based on the underlying studies [21–23]. The study in Mbeya with high estimate had narrow CI (0.109-0161) suggestive of high precision, whereas, one of the two studies in Iringa had a wider CI (0.0997-0.3604) suggestive of low precision. In contrast, the Lake and Western Zones are heavily underrepresented, reflecting uneven research coverage probably due to fewer academic and research institutions in these zones [24]. This calls for coordinated nationwide surveillance to capture true spatial bTB variation.

A conspicuous gap observed by this review is the absence of incidence data, which is in consistent with challenges faced by other low and middle income countries that lack formal herd tracking systems [25]. Only a few of outbreak investigations have been documented in Kagera [26] and drought-related clusters in Iringa [27]. The paucity of outbreak data likely due to chronic progression of the disease, weak surveillance, and unorganized reporting. To address this gap, sentinel herds and longitudinal monitoring need to be established for successful surveillance of the bTB in the country.

This review established that bTB transmission in Tanzania is dictated by a combination of various risk factors categorized into environmental, animal-level, and herd-level. Older animals are more likely to be infected than young animals probably due to prolonged exposure [22]. Similarly, sharing grazing land between neighboring herds and large herd size (a feature in pastoral systems) increased chances of TB transmission [7]. On the other hand, environmental exposure through shared grazing and water sources with wildlife, increased bTB risk. These findings are in congruence with patterns reported in other African countries [28].

Transmission of bTB among cattle occurs directly and indirectly through aerosols [29,30], and contaminated grazing lands and water sources respectively. Reviewed molecular studies revealed similar *M. bovis* strains in cattle and wild animals [7,31], confirming active livestock-wildlife transmission. Although occupational risk among abattoir workers has been adequately reported elsewhere [32], the role of humans in zoonotic transmission is still under researched in Tanzania.

Wildlife animals, for instance African buffalo (*Syncerus caffer*) as *M. bovis* reservoirs maintain bTB, in wildlife ecologies such as Serengeti, Ruaha and Mikumi [8,29]. These reservoirs are vital for bTB transmission to livestock at livestock-wildlife interfaces. The detection of similar spoligotypes in both cattle and buffalo supports evidence for the existence of cross-species transmission. However, other wildlife species such as warthogs, antelopes and giraffes had low infection rates, with small mammals only carrying non-tuberculous mycobacteria [33]. In Tanzania, the role of species like honey badgers, identified as reservoirs in Europe [34,35], remains unstudied. This observation underscores the importance of researching on and monitoring bTB in under studied animals that have been implicated as bTB reservoirs elsewhere.

The review discovered a limited genetic variation of *M. bovis* in Tanzania compared to other African countries. Spoligotype SB0133 dominates livestock and wildlife isolates, with an overall prevalence of 15% across studies. On contrary, Ethiopia is predominated by SB0134 [36], where in Malawi spoligotype SB0131 is reported to be in ubiquitous [37]. Unique strains such as SB2190 and SB2290 further highlight presence of local genetic evolution. Therefore, investing in advanced molecular tools such as whole genome sequencing would enhance clear comprehension of genetic diversity and transmission pathways of the pathogen and the disease respectively.

The Single Intradermal Comparative Tuberculin Test (SCITT) remains the leading research-based diagnostic approach in Tanzania due to its affordability and field applicability features. Nevertheless, its limited sensitivity causes underestimation of prevalence rates in animals with concurrent infections [38]. More specific diagnostic methods such as PCR, and spoligotyping were applied in fewer studies probably due to their high cost and infrastructure availability challenges [39]. These challenges can be overcome by prioritizing affordable rapid diagnostics that are sensitive and user-friendly with respect to field conditions.

Bovine tuberculosis surveillance in Tanzania is almost absent and fragmented, which relies on academic research rather than well-organized government monitoring. Abattoir-based gross lesion records backed with no confirmatory tests are a means of bTB monitoring in Tanzania [40]. Integration of animal and human TB data remains absent or unclear, in spite of its zoonotic significance.

Control measures of bTB in Tanzania are currently limited to meat inspection, movement restrictions, and selective testing [30]. Other proposed control strategies include pilot vaccine testing and adoption of One-Health approaches [27,41]. Developed countries have demonstrated, for the bTB control to be successful robust veterinary services, farmer compensation schemes, and government commitment are key elements to be considered [42]. However, in Tanzania, policies such as test-and-slaughter are yet to be implemented due to financial constraints and lack of farmer compensation schemes [43].

## Conclusions

This review synthesizes available data on bTB epidemiological status in Tanzania to date, spanning through eleven sub-themes across thirty-eight studies. The review discovered data paucity in incidence, outbreak investigation and surveillance. Data on bTB spatial distribution are uneven across regions, with remarkable gaps in the Lake and Western Zones. Also, inclusion of published, English language studies only may have excluded relevant local reports.

The findings point to several implications for policy, One-Health and future research toward successful control of bTB in Tanzania. A multidisciplinary coordinated action with a focus to establishing a national bTB surveillance strategy with integrated livestock-wildlife-human data systems is needed. Improving access to rapid molecular diagnostics would enhance early and reliable detection of the disease, as well as enhanced wildlife surveillance for the role of animal reservoirs in transmission dynamics. Exploring context-appropriate control strategies, such as test-and-slaughter with compensation framework is key to encourage farmer’s compliance. Lastly, bTB should be envisioned in national zoonotic disease control programs under a One-Health umbrella to ensure sustainable and lasting cross-sectoral impact.

## Supporting information

S1 Table Search strategies for each database. **Note:** Detailed search queries used in PubMed, Scopus, AJOL, Google Scholar, and Embase for the systematic review.
(DOCX)

S2 Table Characteristics of included studies. **Note:** Summary of each of the thirty-eight studies included in the review, detailing authors, title, year, region, and target species.
(DOCX)
