# Supplementary material for: Epidemiology of bovine tuberculosis in Tanzania: Systematic review and meta-analysis
Source: PLoS Negl Trop Dis. 2026 Jul 6;20(7):e0014459. doi: 10.1371/journal.pntd.0014459 (PMC13336179; doi:10.1371/journal.pntd.0014459)
Supplement: S1 Table — Note: Detailed search queries used in PubMed, Scopus, AJOL, Google Scholar, and Embase for the systematic review. (DOCX) [file pntd.0014459.s001.docx]

Table S1: Databases searched and their respective search queries

| **Database** | **Search query** | **Results** |
| --- | --- | --- |
| 1. Pubmed | (("bovine tuberculosis" OR "mycobacterium bovis" OR "m. bovis") AND (cattle OR bovine OR livestock OR wildlife) AND (Tanzania OR Zanzibar OR "East Africa")) Filters: English, from 1993 - 2024 | 49 |
| 1. Ajol | ('bovine tuberculosis'/exp OR 'mycobacterium bovis'/exp OR 'm. bovis') AND (cattle OR bovine OR livestock OR animals OR wildlife) AND (tanzania OR 'united republic of tanzania' OR zanzibar OR 'east africa') | Only the first 200 results were screened |
| 1. Scopus | TITLE-ABS-KEY("bovine tuberculosis" OR "Mycobacterium bovis" OR "M. bovis") AND TITLE-ABS-KEY(cattle OR bovine OR livestock OR animals OR wildlife) AND TITLE-ABS-KEY(Tanzania OR "United Republic of Tanzania" OR Zanzibar OR "East Africa") | 55 |
| 1. Embase | ('bovine tuberculosis'/exp OR 'mycobacterium bovis'/exp OR 'm. bovis') AND (cattle OR bovine OR livestock OR animals OR wildlife) AND (tanzania OR 'united republic of tanzania' OR zanzibar OR 'east africa') AND [english]/lim AND [1993-2024]/py | 56 |
| 1. Google scholar | (("bovine tuberculosis" OR "mycobacterium bovis" OR "m. bovis") AND (cattle OR bovine OR livestock OR wildlife) AND (Tanzania OR Zanzibar OR "East Africa"))  • Searched on: 04/07/2025  • Return articles dated between: 1993-2024  • Include no patents  • Include no citations  • Only results from the first 50 google pages were screened | 500 |
