# Supplementary material for: Epidemiology of bovine tuberculosis in Tanzania: Systematic review and meta-analysis
Source: PLoS Negl Trop Dis. 2026 Jul 6;20(7):e0014459. doi: 10.1371/journal.pntd.0014459 (PMC13336179; doi:10.1371/journal.pntd.0014459)
Supplement: S2 Table — Note: Summary of each of the thirty-eight studies included in the review, detailing authors, title, year, region, and target species. (DOCX) [file pntd.0014459.s002.docx]

Table S2: Characteristics of included studies

| **Authors** | **Title** | **Year** | **Region** | **Target** |
| --- | --- | --- | --- | --- |
| Cleaveland et al.[1] | Mycobacterium bovis in rural Tanzania: risk factors for infection in human and cattle populations | 2007 | Manyara | cattle |
| Mwakapujam et al.[2] | Molecular characterization of Mycobacterium bovis isolates from pastoral livestock at Mikumi-Selous ecosystem in the eastern Tanzania | 2013a | Morogoro | cattle and wildlife |
| Kazwala et al.[3] | Risk factors associated with the occurrence of bovine tuberculosis in cattle in the Southern Highlands of Tanzania | 2001 | Iringa and Mbeya | cattle |
| Mwakapuja et al.[4] | Prevalence and significant geospatial clusters of bovine tuberculosis infection at livestock–wildlife interface ecosystem in Eastern Tanzania | 2013b | Morogoro | cattle |
| Shirima et al.[5] | Prevalence of bovine tuberculosis in cattle in different farming systems in the eastern zone of Tanzania | 2003 | Coast and Morogoro | cattle |
| Kazwala et al.[6] | The molecular epidemiology of Mycobacterium bovis infections in Tanzania | 2006 | Mbeya and Arusha and | cattle |
| Mugambi et al.[7] | Bovine tuberculosis in East Africa | 2016 | Southern highlands zone (SHZ), eastern zone (EZ) and northern zone (NZ) | cattle |
| Cleaveland et al.[8] | Tuberculosis in Tanzanian wildlife | 2005 | Arusha (Northen national parks) | wildlife |
| Travis et al.[9] | Environmental reservoirs of Mycobacterium bovis and Mycobacterium tuberculosis in the Ruaha region, Tanzania | 2019 | Iringa | cattle, soil, dust |
| Weinhäupl et al.[10] | Investigations on the prevalence of bovine tuberculosis and brucellosis in dairy cattle in Dar es Salaam region and in zebu cattle in Lugoba area, Tanzania | 2000 | Dar es salaam | cattle |
| Roug et al.[11] | Spatial predictors of bovine tuberculosis infection and Brucella spp. exposure in pastoralist and agropastoralist livestock herds in the Ruaha ecosystem of Tanzania | 2014 | Iringa | cattle |
| Clifford et al.[12] | Tuberculosis infection in wildlife from the Ruaha ecosystem Tanzania: implications for wildlife, domestic animals, and human health | 2013 | Iringa | wildlife |
| Swai & Schoonman[13] | Differences in prevalence of tuberculosis in indigenous and crossbred cattle under extensive and intensive management systems in Tanga Region of Tanzania | 2011 | Tanga | cattle |
| Durnez et al.[14] | Mycobacteria in terrestrial small mammals on cattle farms in Tanzania | 2011 | Morogoro | cattle and wildlife |
| Berg et al.[15] | African 2, a Clonal Complex of Mycobacterium bovis Epidemiologically Important in East Africa | 2011 | Not specified | cattle |
| Kazwala et al.[16] | Prevalence of bovine tuberculosis in human and cattle in rift valley districts of Arusha region. | 1999 | Arusha | cattle |
| Komba et al.[17] | Sanitary practices and occurrence of zoonotic conditions in cattle at slaughter in Morogoro Municipality, Tanzania: implications for public health | 2012 | Morogoro | cattle |
| Mwakapuja et al.[18] | Detection of bovine tuberculosis in African buffaloes and indigenous cattle in Mikumi ecosystem, Tanzania | 2014 | Morogoro | wildlife |
| Katale et al.[19] | Isolation and Potential for Transmission of Mycobacterium bovis at Human-livestock-wildlife Interface of the Serengeti Ecosystem, Northern Tanzania | 2017 | Arusha | cattle and wildlife |
| Katale et al.[20] | Screening for bovine tuberculosis in African buffalo (syncerus caffer) in Ngorongoro conservation area, Northern tanzania: Implications for public health | 2017 | Arusha | wildlife |
| Jiwa et al.[21] | Bovine tuberculosis in the Lake Victoria zone of Tanzania and its possible consequences for human health in the HIV/AIDS era | 1997 | Lake zone | cattle |
| Roug et al.[22] | Health of African Buffalos (Syncerus caffer) in Ruaha National Park, Tanzania | 2020 | Iringa | wildlife |
| Karimuribo et al.[23] | Studies on mastitis, milk quality and health risks associated with consumption of milk from pastoral herds in Dodoma and Morogoro regions, Tanzania | 2005 | Dodoma and Morogoro | cattle |
| Mdegela et al.[24] | Prevalence and determinants of mastitis and milk-borne zoonoses in smallholder dairy farming sector in Kibaha and Morogoro districts in Eastern Tanzania | 2004 | Coast and Morogoro | cattle |
| Swai & Schoonman [25] | A survey of zoonotic diseases in trade cattle slaughtered at Tanga city abattoir: A cause of public health concern | 2012 | Tanga | cattle |
| Mdegela et al.[26] | Mastitis in smallholder dairy and pastoral cattle herds in the urban and peri-urban areas of the Dodoma municipality in Central Tanzania | 2005 | Dodoma | cattle |
| Villa et al.[27] | "One Health" approach to end zoonotic TB | 2023 | Not specified | NA |
| Katale et al.[28] | Prevalence and risk factors for infection of bovine tuberculosis in indigenous cattle in the Serengeti ecosystem, Tanzania. | 2013 | Arusha | cattle |
| Durnez et al.[29] | The prevalence of Mycobacterium bovis-infection and atypical mycobacterioses in cattle in and around Morogoro, Tanzania. | 2009 | Morogoro | cattle |
| Kazwala et al.[30] | Isolation of Mycobacterium species from raw milk of pastoral cattle of the Southern Highlands of Tanzania | 1998 | Iringa and Mbeya | cattle |
| Mariki et al.[31] | Prevalence of Bovine Tuberculosis in indigenous cattle in Gairo district of Morogoro Region in Tanzania | 2013 | Morogoro | catte |
| Mkombozi et al.[32] | Prevalence and Risk factors of Bovine Tuberculosis in smallholder dairy cattle in Babati town council of Manyara region, Tanzania | 2014 | Manyara | cattle |
| Medardus[33] | Bovine tuberculosis and brucellosis in Livestock at the Greater Ruaha Ecosystem | 2020 | Iringa | cattle and goats |
| Kazwala et al.[34] | The prevalence of bovine tuberculosis in Rift Valley Districts based on single intradermal comparative tuberculin testing | 2000 | Arusha | cattle |
| Durnez et al.[35] | First detection of mycobacteria in African rodents and insectivores, using stratified pool screening | 2008 | Morogoro | wildlife |
| Kambarage et al.[36] | Disease conditions responsible for condemnation of carcasses and organs in short-horn Zebu cattle slaughtered in Tanzania. | 1995 | Morogoro | catte |
| Mellau et al.[37] | A slaughterhouse survey of liver lesions in slaughtered cattle, sheep and goats at Arusha, Tanzania | 2010 | Arusha | cattle |
| Mellau et al.[38] | Slaughter stock abattoir survey of carcasses and organ/offal condemnations in Arusha Region, northern Tanzania. | 2011 | Arusha | cattle |

**References**

[1] S. Cleaveland *et al.*, “Mycobacterium bovis in rural Tanzania: risk factors for infection in human and cattle populations.,” *Tuberculosis (Edinb).*, vol. 87, no. 1, pp. 30–43, Jan. 2007, doi: 10.1016/j.tube.2006.03.001.

[2] R. S. Mwakapuja, Z. E. Makondo, J. Malakalinga, I. Moser, R. R. Kazwala, and M. Tanner, “Molecular characterization of Mycobacterium bovis isolates from pastoral livestock at Mikumi-Selous ecosystem in the eastern Tanzania.,” *Tuberculosis (Edinb).*, vol. 93, no. 6, pp. 668–674, Nov. 2013, doi: 10.1016/j.tube.2013.08.002.

[3] R. R. Kazwala *et al.*, “Risk Factors Associated with the occurrence of Bovine tuberculosis in cattle in the Southern Highlands of Tanzania,” *Vet. Res. Commun.*, vol. 25, no. 8, pp. 609–614, 2001.

[4] R. S. Mwakapuja *et al.*, “Prevalence and significant geospatial clusters of bovine tuberculosis infection at livestock – wildlife interface ecosystem in Eastern Tanzania,” *1224 Trop Anim Heal. Prod*, vol. 45, pp. 1223–1230, 2013, doi: 10.1007/s11250-013-0350-2.

[5] G. M. Shirima, R. R. Kazwala, and D. M. Kambarage, “Prevalence of bovine tuberculosis in cattle in different farming systems in the eastern zone of Tanzania,” *Prev. Vet. Med.*, vol. 57, no. 3, pp. 167–172, 2003, doi: 10.1016/S0167-5877(02)00214-3.

[6] R. R. Kazwala, L. J. M. Kusiluka, K. Sinclair, J. M. Sharp, and C. J. Daborn, “The molecular epidemiology of Mycobacterium bovis infections in Tanzania.,” *Vet. Microbiol.*, vol. 112, no. 2–4, pp. 201–210, Feb. 2006, doi: 10.1016/j.vetmic.2005.11.026.

[7] J. M. Mugambi *et al.*, “Bovine tuberculosis in East Africa,” *African Crop Sci. J.*, vol. 24, no. s1, pp. 53–61, 2016, doi: http://dx.doi.org/10.4314/acsj.v24i1.6S BOVINE.

[8] S. Cleaveland *et al.*, “Tuberculosis in Tanzanian wildlife,” *J. Wildl. Dis.*, vol. 41, no. 2, pp. 446–453, 2005, doi: https://doi.org/10.7589/0090-3558-41.2.446.

[9] E. R. Travis *et al.*, “Environmental reservoirs of Mycobacterium bovis and Mycobacterium tuberculosis in the Ruaha region, Tanzania,” *bioRxiv*, pp. 1–29, 2019, doi: doi: https://doi.org/10.1101/790824.

[10] I. Weinhäupl, K. C. Schöpf, D. Khaschabi, A. M. Kapaga, and H. M. Msami, “Investigations on the prevalence of bovine tuberculosis and brucellosis in dairy cattle in Dar es Salaam region and in zebu cattle in Lugoba area, Tanzania,” *Trop. Anim. Health Prod.*, vol. 32, no. 3, pp. 147–154, 2000, doi: 10.1023/A:1005231514467.

[11] A. Roug *et al.*, “Spatial predictors of bovine tuberculosis infection and Brucella spp. exposure in pastoralist and agropastoralist livestock herds in the Ruaha ecosystem of Tanzania.,” *Trop. Anim. Health Prod.*, vol. 46, no. 5, pp. 837–843, Jun. 2014, doi: 10.1007/s11250-014-0574-9.

[12] D. L. Clifford *et al.*, “Tuberculosis infection in wildlife from the Ruaha ecosystem Tanzania: implications for wildlife, domestic animals, and human health.,” *Epidemiol. Infect.*, vol. 141, no. 7, pp. 1371–1381, Jul. 2013, doi: 10.1017/S0950268813000836.

[13] E. S. Swai and L. Schoonman, “Differences in prevalence of tuberculosis in indigenous and crossbred cattle under extensive and intensive management systems in Tanga region of Tanzania.,” *Trop. Anim. Health Prod.*, vol. 44, no. 3, pp. 459–465, Mar. 2012, doi: 10.1007/s11250-011-9919-9.

[14] L. Durnez *et al.*, “Mycobacteria in terrestrial small mammals on cattle farms in Tanzania,” *Vet. Med. Int.*, vol. 2011, 2011, doi: 10.4061/2011/495074.

[15] S. Berg *et al.*, “African 2, a clonal complex of Mycobacterium bovis epidemiologically important in East Africa.,” *J. Bacteriol.*, vol. 193, no. 3, pp. 670–678, Feb. 2011, doi: 10.1128/JB.00750-10.

[16] G. Kazwala, R.R., Sharp, J.M., Mfinanga, G., Kunda, J., Cleaveland, S. and Shirima, “Prevalence of Bovine Tuberculosis in Human and Cattle in Rift Valley Districts of Arusha Region,” *Tanzania Vet. J.*, pp. 642–646, 1999.

[17] E. V. G. Komba *et al.*, “Sanitary practices and occurrence of zoonotic conditions in cattle at slaughter in Morogoro Municipality, Tanzania: implications for public health.,” *Tanzan. J. Health Res.*, vol. 14, no. 2, pp. 131–138, Apr. 2012, doi: 10.4314/thrb.v14i2.6.

[18] R. S. Mwakapuja *et al.*, “Detection of bovine tuberculosis in African buffaloes and indigenous cattle in Mikumi ecosystem, Tanzania,” *Tanzania Vet. J.*, vol. 29, no. 2, pp. 35–42, 2014.

[19] B. Z. Katale *et al.*, “Isolation and Potential for Transmission of Mycobacterium bovis at Human-livestock-wildlife Interface of the Serengeti Ecosystem, Northern Tanzania.,” *Transbound. Emerg. Dis.*, vol. 64, no. 3, pp. 815–825, Jun. 2017, doi: 10.1111/tbed.12445.

[20] B. Z. Katale *et al.*, “Screening for bovine tuberculosis in African buffalo (Syncerus caffer) in ngorongoro conservation area, northern Tanzania: Implications for public health,” *J. Wildl. Dis.*, vol. 53, no. 4, pp. 711–717, 2017, doi: 10.7589/2016-10-223.

[21] S. F. H. Jiwa, A. A. O. Aboud, R. R. Kazwala, and W. J. Kalaye, “Bovine tuberculosis in the Lake zone of Tanzania and possible consequences for human health in the HIV / AIDS era,” *Vet. Res. Commun.*, vol. 21, no. 8, pp. 533–539, 1997.

[22] A. Roug *et al.*, “Health of African Buffalos (Syncerus caffer) in Ruaha National Park, Tanzania.,” *J. Wildl. Dis.*, vol. 56, no. 2, pp. 495–498, Apr. 2020, doi: 10.7589/2019-06-151.

[23] E. D. Karimuribo, L. J. Kusiluka, R. H. Mdegela, A. M. Kapaga, C. Sindato, and D. M. Kambarage, “Studies on mastitis, milk quality and health risks associated with consumption of milk from pastoral herds in Dodoma and Morogoro regions, Tanzania.,” *J. Vet. Sci.*, vol. 6, no. 3, pp. 213–221, Sep. 2005.

[24] R. H. Mdegela *et al.*, “Prevalence and determinants of mastitis and milk-borne zoonoses in smallholder dairy farming sector in Kibaha and Morogoro districts in Eastern Tanzania.,” *J. Vet. Med. B, Infect. Dis. Vet. public Heal.*, vol. 51, no. 3, pp. 123–128, Apr. 2004, doi: 10.1111/j.1439-0450.2004.00735.x.

[25] E. S. Swai and L. Schoonman, “A survey of zoonotic diseases in trade cattle slaughtered at Tanga city abattoir: A cause of public health concern,” *Asian Pac. J. Trop. Biomed.*, vol. 2, no. 1, pp. 55–60, 2012, doi: 10.1016/S2221-1691(11)60190-1.

[26] R. H. Mdegela *et al.*, “Mastitis in smallholder dairy and pastoral cattle herds in the urban and peri-urban areas of the Dodoma municipality in Central Tanzania,” *Livest. Res. Rural Dev.*, vol. 17, no. 11, p. 123, 2005.

[27] S. Villa *et al.*, “’One Health´ approach to end zoonotic TB.,” *Int. J. Tuberc. lung Dis. Off. J. Int. Union against Tuberc. Lung Dis.*, vol. 27, no. 2, pp. 101–105, Feb. 2023, doi: 10.5588/ijtld.22.0393.

[28] B. Z. Katale *et al.*, “Prevalence and risk factors for infection of bovine tuberculosis in indigenous cattle in the Serengeti ecosystem, Tanzania,” *BMC Vet. Res.*, vol. 9, no. 267, pp. 1–11, 2013, doi: 10.1186/1746-6148-9-267.

[29] L. Durnez *et al.*, “The prevalence of Mycobacterium bovis-infection and atypical mycobacterioses in cattle in and around Morogoro, Tanzania.,” *Trop. Anim. Health Prod.*, vol. 41, no. 8, pp. 1653–1659, Dec. 2009, doi: 10.1007/s11250-009-9361-4.

[30] R. R. Kazwala, C. J. Daborn, L. J. M. Kusiluka, S. F. H. Jiwa, J. M. Sharp, and D. M. Kambarage, “Isolation of Mycobacterium species from raw milk of pastoral cattle of the Southern Highlands of Tanzania,” *Trop. Anim. Health Prod.*, vol. 30, no. 4, pp. 233–239, 1998, doi: 10.1023/A:1005075112393.

[31] M. B. Mariki, E. D. Karimuribo, and R. R. Kazwala, “Prevalence of Bovine Tuberculosis in indigenous cattle in Gairo district of Morogoro Region in Tanzania,” *Tanzania Vet. J.*, vol. 28, no. 1, pp. 51–59, 2013, doi: 10.4314/tvj.v28i1.

[32] F. A. Mkombozi, R. R. Kazwala, and A. M. Lupindu, “Prevalence and Risk factors of Bovine Tuberculosis in smallholder dairy cattle in Babati town council of Manyara region, Tanzania,” *Tanzania Vet. J.*, vol. 29, no. 1, pp. 30–38, 2014

[33] J. J. Medardus, “Bovine tuberculosis and brucellosis in Livestock at the Greater Ruaha Ecosystem,” *Tanzania Vet. J. Vol.*, vol. 35, no. 1, pp. 63–80, 2020.

[34] R. R. Kazwala *et al.*, “The prevalence of bovine tuberculosis in Rift Valley Districts based on single intradermal comparative tuberculin testing.,” *Tanzania Vet. J.*, vol. 20, pp. 136–140, 2000, [Online].

[35] L. Durnez *et al.*, “First detection of mycobacteria in African rodents and insectivores, using stratified pool screening.,” *Appl. Environ. Microbiol.*, vol. 74, no. 3, pp. 768–773, Feb. 2008, doi: 10.1128/AEM.01193-07.

[36] D. M. Kambarage, S. I. Kimera, R. R. Kazwala, and B. M. Mafwere, “Disease conditions responsible for condemnation of carcasses and organs in short-horn Zebu cattle slaughtered in Tanzania,” *Prev. Vet. Med.*, vol. 22, no. 4, pp. 249–255, 1995, doi: 10.1016/0167-5877(94)00421-E.

[37] L. S. . Mellau, H. . Nonga, and E. . Karimuribo, “A slaughterhouse survey of liver lesions in slaughtered cattle, sheep and goats at Arusha, Tanzania,” *Res. J. Vet. Sci.*, vol. 3, no. 3, pp. 179–188, 2010

[38] B. L. Mellau, H. E. Nonga, and E. D. Karimuribo, “Slaughter stock abattoir survey of carcasses and organ/offal condemnations in Arusha region, northern Tanzania.,” *Trop. Anim. Health Prod.*, vol. 43, no. 4, pp. 857–864, Apr. 2011, doi: 10.1007/s11250-010-9773-1.
